# Comparative study of SSVEP characteristics in mixed versus virtual reality across varying depths

**DOI:** 10.3389/fnins.2026.1713018

**Published:** 2026-02-27

**Authors:** Qian Zhang, Zehui Cao, Songling Tian, Zhuoke Cai, Liwen Shi, Xiaoqian Qi

**Affiliations:** 1 School of Control and Mechanical Engineering, Tianjin Chengjian University, Tianjin, China; 2 School of Electrical and Information Engineering, Tianjin University, Tianjin, China; 3 Tianjin Ren’ai College, Tianjin, China

**Keywords:** brain-computer interface (BCI), depth stimuli, mixed reality (MR), steady-state visual evoked potential (SSVEP), vergence-accommodation conflict (VAC)

## Abstract

Steady-state visually evoked potentials (SSVEP), owing to their high signal-to-noise ratio and low training cost, are widely regarded as an effective approach for constructing visually driven brain-computer interfaces (BCI), particularly in neurorehabilitation applications. However, the accommodation-vergence conflict (VAC) commonly present in mixed reality (MR) and virtual reality (VR) head-mounted displays may attenuate neural responses in the visual cortex, thereby compromising the long-term usability of such systems. This study aims to systematically evaluate the effects of MR and VR environments under different virtual depth conditions on SSVEP signal quality, classification performance, and visual comfort, providing parameter guidelines for the design of immersive visual BCIs in rehabilitation contexts. Green flickering stimuli at 7.5, 11.25, and 18 Hz were presented at three virtual depths of 0.4, 1.0, and 1.8 m. Feature extraction and classification were performed using canonical correlation analysis (CCA), Filter-Bank Canonical Correlation Analysis (FBCCA), and task-related component analysis (TRCA).The results showed a negative correlation between stimulus distance and SSVEP classification accuracy, with FBCCA achieving the highest accuracy at the 0.4 m depth (71.8% ± 33.8%). Overall, the signal-to-noise ratio (SNR) in the MR environment was higher than that in the VR environment, with the most pronounced difference observed under the 1.8 m condition, suggesting that MR is more effective in alleviating VAC and maintaining stable visual cortical responses. Among the three stimulation frequencies, 11.25 Hz elicited the highest SSVEP amplitude and SNR, indicating it as the optimal frequency band. Subjective visual fatigue assessments revealed higher scores for VR in terms of diplopia and fixation difficulty, with trends consistent with the observed SNR reduction. This study elucidates the interactive modulation effects of virtual depth, display modality, and flicker frequency on SSVEP, and demonstrates that MR outperforms VR in terms of signal stability, visual comfort, and potential rehabilitation usability. The derived parameters provide experimentally validated optimization strategies for stimulus depth and frequency in vision-based attention training, spatial orientation training, upper-limb interactive tasks, and immersive feedback systems in neurorehabilitation, thereby contributing to improved long-term adherence and clinical translational value of future rehabilitation BCI.

## 1 Introduction

Brain-computer interface (BCI) is a technology that establishes a direct communication pathway between the brain and external device. By decoding signals produced by central nervous system activity, a BCI enables users to control external devices (Tao et al., 2023, Duart et al., 2020). This method provides a novel assistive approach for patients with motor impairments and central nervous system disorders. The core mechanism is the real-time collection and decoding of brain signals, translating the user’s intended movements into control commands to drive external devices (e.g., moving a robotic arm or controlling a cursor). This “thought-to-action” direct mapping allows users to interact with their environment in a nearly natural way, improving autonomy in daily life (Hochberg et al., 2012; Bouton et al., 2016). BCIs can be categorized into invasive and non-invasive types based on the signal acquisition method. Invasive BCIs require implanting electrodes in the cerebral cortex to obtain high-SNR neural signals (SNR > 20 dB), but this approach entails surgical risks (for example, approximately 4.7% risk of intracranial hemorrhage) (Oxley, 2022). Non-invasive BCIs record signals such as scalp electroencephalography (EEG), offering much higher safety but lower signal resolution due to skull attenuation (spatial resolution > 1 cm), which often limits decoding accuracy (Huang et al., 2019).

Among the various non-invasive paradigms, steady-state visual evoked potentials (SSVEPs) have attracted considerable attention due to their high signal-to-noise ratio (SNR) and excellent information transfer rate (ITR). This advantage can be attributed to the phase-locked nature of the neural response to periodic visual stimuli. Typically, the SNR of SSVEP signals is 3–5 times higher than that of motor imagery paradigms (Chen et al., 2015), and the integration of multiple harmonics can further improve signal quality (Bin et al., 2009). When combined with sophisticated algorithms (e.g., FBCCA), SSVEP-based BCI systems have attained information transfer rates exceeding 120 bits/min (Chen et al., 2015), significantly surpassing those of other non-invasive methods. SSVEPs refer to the periodic electroencephalogram (EEG) responses elicited by visual stimuli that flash at fixed frequencies (Park et al., 2021). The neural activity of these cells originates in the primary visual cortex and manifests as oscillatory signals that are phase-locked to the stimulus frequency and its harmonics (Wittevrongel and Van Hulle, 2016). When a user fixates on a flickering stimulus, the visual cortex generates neural oscillations at the stimulus’s fundamental frequency (*F*_0_) and harmonic components (2*F*_0_, 3*F*_0_, etc.). The energy of these responses is consistent with a resonance model, producing stable spectral peaks at the corresponding frequencies over occipital electrodes (Norcia et al., 2015).

Traditional SSVEP-based BCI typically employ a spatially coded frequency-mapping paradigm, wherein multiple stimulus sources flashing at different frequencies are used for command selection (Cinel et al., 2019). LCD displays in such systems must have optimized refresh rates to reduce visual fatigue, whereas LED arrays can provide stronger harmonic responses. However, LCD-based SSVEP systems still face two major limitations: Stimulus frequency constraints: The fixed refresh rate of the display limits the choice of stimulus frequencies, as dictated by the principles of discrete-time sampling on digital displays (Nakanishi et al., 2014); User visual comfort: Strong flickering causes visual discomfort. Reducing the modulation depth (MD = 0.5) can maintain approximately 80% classification in accuracy while alleviating visual fatigue (Gilford et al., 2024). To expand control commands, the dual-frequency dual-phase modulation method (Liang et al., 2020) enables targeting of numerous points by varying combinations of two frequencies and initial phases. A rapid optimization method for designing dual-frequency and dual-phase modulation stimulus codes was developed, achieving an accuracy of 96.06 ± 4.00% and an average information transfer rate (ITR) of 196.09 ± 15.25 bits. This significantly surpasses existing dual-frequency modulation paradigms. For isolating environmental interference, spatial filtering algorithms such as Task-Related Component Analysis (TRCA) (Nakanishi et al., 2018) and Canonical Correlation Analysis (CCA)-based methods (Lin et al., 2006) have been widely adopted to enhance SNR by extracting task-related components and suppressing background noise. Both TRCA and FBCCA methods enhance SNR, though through different approaches. FBCCA decomposes EEG signals into multiple subbands using filters, calculates typical correlations with stimulus frequencies (including harmonics) within each subband, and then integrates subband information via a weighted fusion strategy. This mechanism effectively separates signal from noise, thereby significantly improving the signal-to-noise ratio of SSVEP signals.

Calibration-free SSVEP decoding methods have emerged as a critical research direction in recent years, aiming to eliminate the time-consuming subject-specific calibration process and improve the practicality of BCI systems—especially for clinical rehabilitation and rapid deployment scenarios. Traditional SSVEP decoding (e.g., CCA, FBCCA) relies on pre-collected calibration data to construct reference templates or spatial filters, which limits usability for patients with limited attention spans or urgent clinical needs (Wong et al., 2022). Calibration-free methods address this limitation by leveraging generic templates, online iterative learning, or cross-subject transfer learning, with key advances as follows:

1. Generic template-based methods: These methods construct universal reference templates using data from a large number of subjects, avoiding individual calibration. For example, Nakanishi et al. (2015) proposed a generic CCA template library, but its performance degrades significantly for subjects with atypical SSVEP responses due to inter-subject variability.

2. Online iterative learning methods: By updating decoding models in real time during use, these methods achieve “zero pre-calibration” while gradually adapting to individual characteristics. Wong et al. (2022) proposed Online Adaptive CCA (OACCA), which initializes with generic templates and iteratively updates weights using newly collected trial data, achieving 78.3% accuracy for 3-class SSVEP decoding without pre-calibration. Building on this, Jin et al. (2024) developed Sparse Transferable Ensemble OACCA (STE-OACCA), integrating sparse regularization and cross-subject transfer learning to enhance adaptation speed—reducing the convergence time by 40% compared to OACCA while maintaining 81.2% accuracy.

3. Advanced decoding baselines based on large-scale pre-training: This category represents another frontier in current SSVEP decoding research. Its core approach involves training powerful deep models or complex feature extractors using massive datasets, then transferring this “expert model” to new users. Typically, only a minimal amount of data (few samples) is required for fine-tuning or adaptation. For instance, the deep transfer learning framework proposed by Ke et al. (2024) pre-trains deep networks on large-scale datasets and then aligns the distribution of new user data through domain adaptation (e.g., MMD), achieving performance comparable to calibrated methods. Jin et al. (2025) take this further with their CSTLF-CPRC framework, incorporating complex spatio-temporal learning modules and probabilistic classifiers to achieve exceptionally high zero-shot or few-shot recognition accuracy on new users after pre-training. Although these methods rely on computationally expensive offline pre-training, they represent the upper performance limit achievable under abundant data conditions, providing crucial performance benchmarks for evaluating other lightweight approaches.

Despite these advances, calibration-free methods still face challenges in immersive environments (MR/VR): few studies have validated their performance across different virtual depths and display modes (MR and VR).

With recent advancements in immersive technologies have prompted researchers to explore the integration of virtual reality (VR) and augmented reality (AR) with SSVEP-BCIs (steady-state visual evoked potential brain-computer interfaces) (Choi et al., 2019). This integration aims to enhance the naturalness of the interaction experience and the system’s immersion capacity. A thorough review indicated the considerable potential of integrating virtual reality (VR) with SSVEP-BCI to enhance usability (Frutos-Pascual et al., 2025). Research in the field of electroencephalogram (EEG)-based brain-computer interfaces has recently shown a growing trend, particularly in applications within immersive virtual reality and augmented reality scenarios (Nwagu et al., 2023). In the domain of augmented reality (AR), researchers have employed SSVEP-BCI technology in conjunction with optical see-through headsets, such as HoloLens. The integration of SSVEP-BCI with mixed reality (MR) has been demonstrated to yield recognition accuracies of approximately 87.7%, 95.4%, and 97.6% for data lengths of 1, 1.5, and 2 s, respectively. The corresponding information transfer rates (ITR) were approximately 64.6, 62.9, and 55.6 bits/min (Liu et al., 2019). Recent studies have investigated the impact of stereoscopic vision (3D stimuli) on the performance of the VR-SSVEP system and the user experience. The findings of the present study indicate that the incorporation of three-dimensional stimuli, such as 3D spheres, 3D flashing lights, or 3D checkerboards, within the context of VR environments results in a substantial enhancement of user experience and classification performance within the mid-frequency range (Liu et al., 2024). Existing research indicates that significant progress has been made in the field of VR/MR-BCI technology. In the context of virtual reality, distance perception is frequently systematically underestimated. This phenomenon is closely related to the convergence-accommodation conflict, thereby affecting visual comfort and neural signal stability (Creem-Regehr et al., 2023).

**FIGURE 1:**
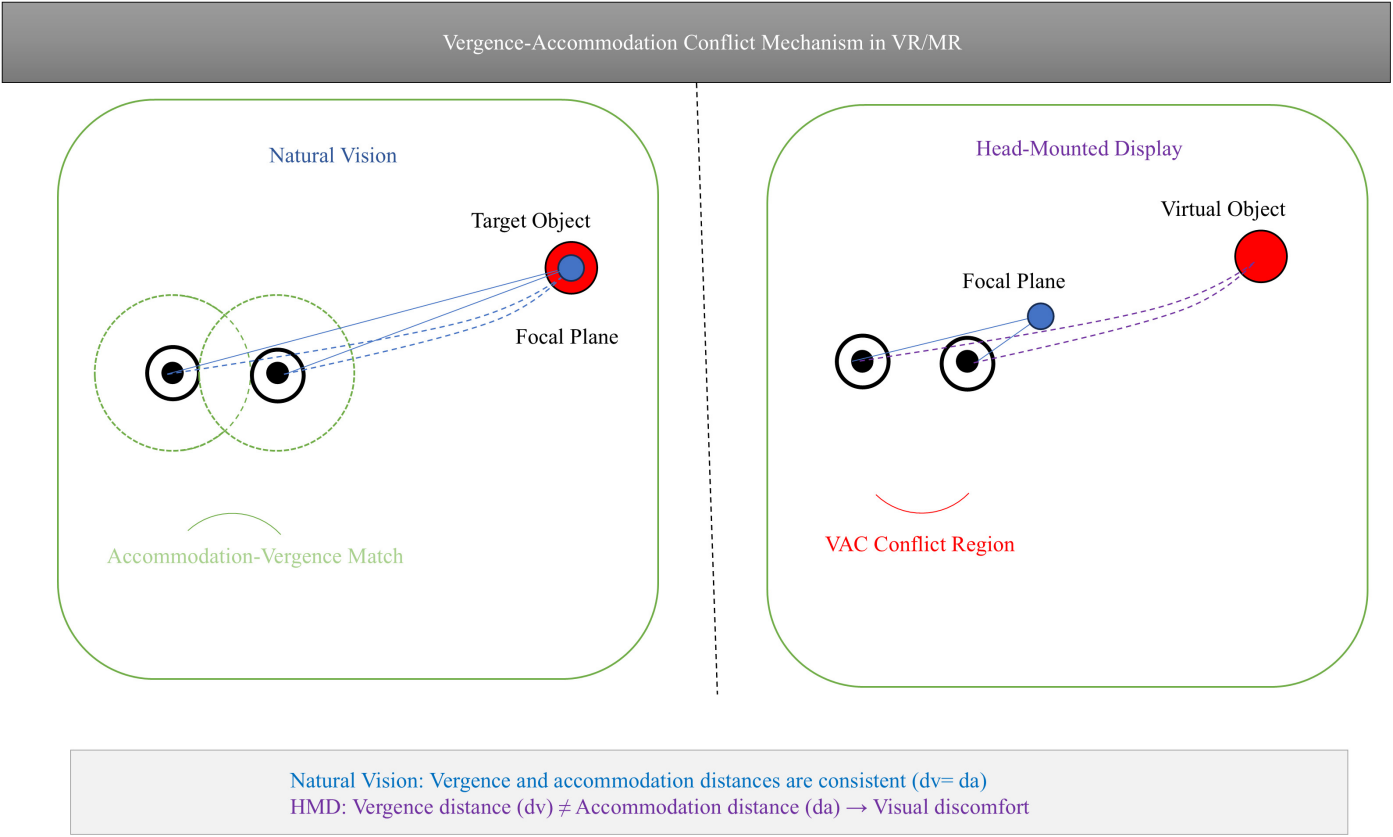
Stimulus difference map between natural vision and head-mounted displays.

However, despite the possibilities that VR/MR bring to BCIs, the common vergence-accommodation conflict (VAC) problem in head-mounted displays (HMDs) still limits SSVEP signal stability and user comfort (Zhou et al., 2021). For patients diagnosed with movement disorders who require long-term, comfortable use of the device, reducing fatigue caused by VAC is of critical importance, as it directly impacts the system’s practical usability and widespread adoption. Accommodation is defined as the eye’s lens changing curvature to achieve a clear image, while vergence is the coordinated rotation of both eyes to maintain a unified binocular view. When an object is observed at a nearby distance, the accommodation of the eyes increases, resulting in a more convex lens shape. Concurrently, the eyes’ capacity for focusing also undergoes enhancement. Conversely, when an object is observed at a distant distance, the lens becomes more flat, and the eyes diverge (Creem-Regehr et al., 2023). Under normal physiological conditions, visual accommodation and visual convergence exhibit a strong coupling relationship, which is regulated by the collaborative action of the visual cortex and the oculomotor nuclei in the brain. The mismatch between the fixed screen of VR/MR headsets (e.g., 2 cm) and the focal length set by the Apple Vision Pro optical system (e.g., approximately 2 m) and its variable virtual depth (e.g., 0.4–1.8 m) triggers VAC. When virtual stimuli emerge at 0.4 m (in close proximity to the focal length), the eye’s accommodation system persists in its attempt to focus on the 2.0 m screen, while the convergence system is compelled to rotate inward to align with the 0.4 m virtual image. This results in a mismatch in neural signals between the components of the visual system, leading to VAC. As demonstrated in Figure 1: stimulus difference map between natural vision and head-mounted displays. This mismatch instigates VAC, whereby the accommodation system remains fixed at the screen plane while vergence follows the virtual depth. This phenomenon leads to desynchronization of the visual system (Kramida, 2015). This conflict has been shown to induce visual fatigue, leading to a decrease in SSVEP amplitude and a downward trend in SNR (Azadi Moghadam and Maleki, 2023).

In order to surmount this impediment, the present study introduces the integration of MR spatial localization with multi-depth SSVEP paradigms. The employment of Apple Vision Pro facilitates the overlaying of virtual stimuli within real-world environments, with its eye-tracking capabilities enabling the precise analysis of fixation points (Plopski et al., 2022). The device adopts a pancake folded optical design to shorten the screen-lens distance and incorporates dynamic rendering algorithms, which can reduce visual fatigue. Building on this capability, we designed target stimuli at three different depths in 3D space (0.4, 1.0, 1.8 m), using green flickering stimuli at 7.5, 11.25, and 18 Hz (wavelength 510–550 nm). This wavelength was chosen because it maintains a sufficient SSVEP response amplitude while reducing the risk of triggering epileptic seizures (Parra et al., 2007). By comparing SSVEP characteristics under different depth conditions and in MR versus VR scenarios, this study aims to explore a BCI interaction mode that more closely mirrors real-world visual experience.

## 2 Experimental design and methods

### 2.1 Participants

The present study recruited ten healthy subjects with normal vision (five males and five females, aged 23 ± 3 years). Prior to the commencement of the experiment, all participants were thoroughly apprised of the study’s objectives, procedures, and potential post-experimental outcomes, and provided written informed consent. The present study received ethical approval from the Tianjin University Ethics Committee (approval number TJUE-2025-210 dated 26 February 2025).

### 2.2 Experimental apparatus and stimuli

**FIGURE 2:**
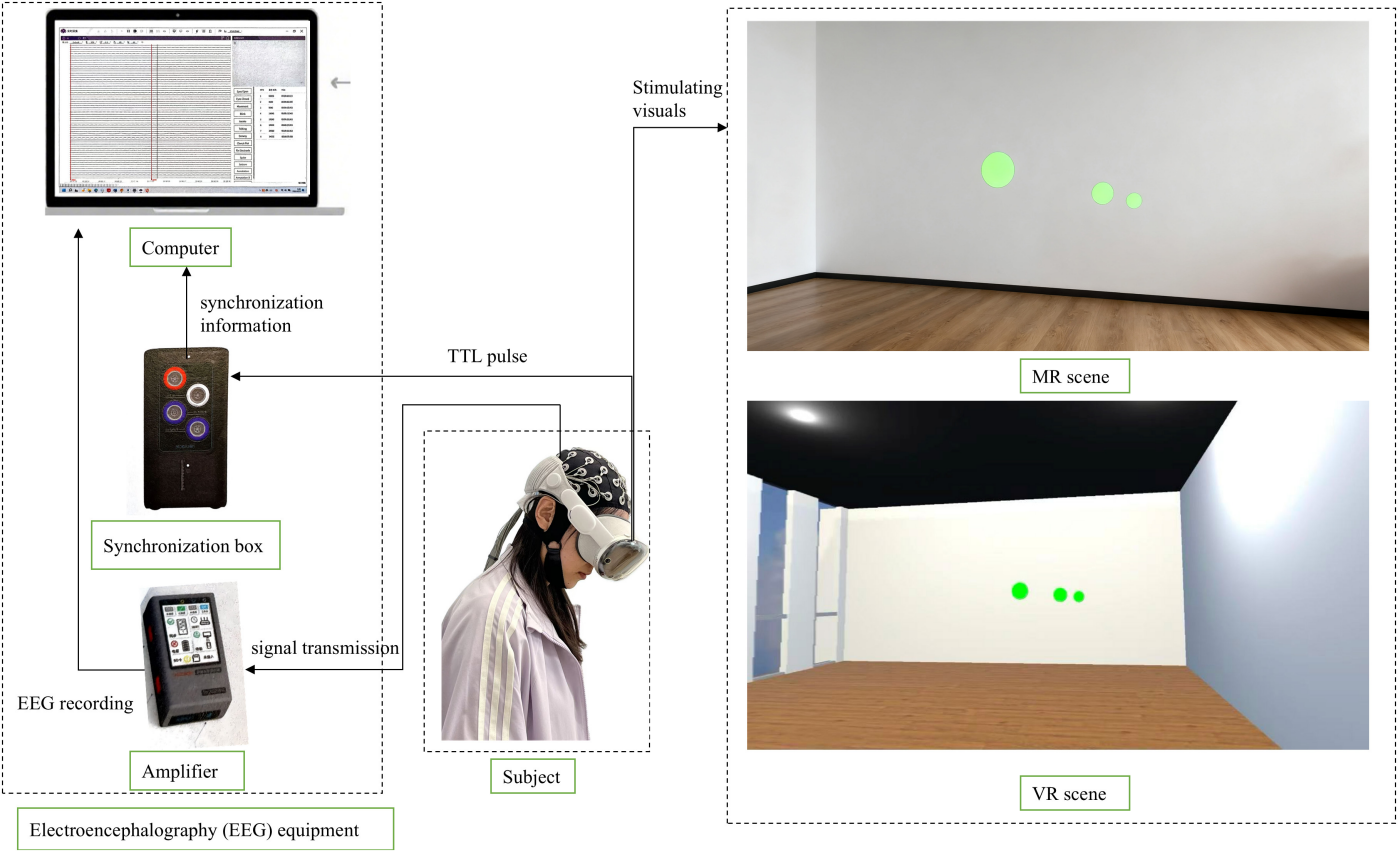
Data collection process.

The experiment utilized an Apple Vision Pro headset in conjunction with a Neuracle wearable multimodal EEG cap. Developed a virtual scene in Unity 3D that replicated the real environment, and deployed this VR scene with the flickering stimuli onto the Vision Pro, thereby enabling both MR and VR experimental conditions. EEG data were recorded in real time using NeuroHub software. A multi-modal data acquisition hub integrated various sensor inputs and transmitted them to the recording software. Meanwhile, a synchronization unit was connected to the computer via a serial port to send event markers, and the EEG amplifier performed clock synchronization across all data streams and transmitted the signals to the acquisition software. The acquisition computer recorded all synchronized data. The data collection process is shown in Figure 2.

The stimulus application was created in Unity 3D and run on the Vision Pro (uploaded via USB). The stimuli were driven at a fixed display refresh rate of 90 Hz (i.e., the screen updates 90 frames per second). According to the principles of discrete-time sampling and reconstruction, a flicker period must consist of an integer number of frames under the display’s refresh constraint (Nakanishi et al., 2014), which avoids any fractional-frame timing errors and simplifies precise timing implementation.

We designed flickering targets at three different viewing distances: 0.4, 1.0, and 1.8 m from the user. The 0.4 m distance represents a near-field, fine interaction (such as reading or operating a handheld device). At this distance, stereoscopic vision will trigger a convergence-accommodation conflict, which is a sensitive area for visual discomfort (Shibata et al., 2011). Previous near-field AR depth-matching experiments often used a range of 33–50 cm to represent near tasks, so 0.4 m was a reasonable choice for the near distance. At close viewing distances, the demand on the eyes’ accommodation is highest, and the vergence-accommodation conflict (VAC) is especially pronounced. VAC is a core issue with head-mounted displays for near tasks, leading to visual fatigue and fusion difficulty (this has been documented in detailed reviews and assessments). Therefore, the 0.4 m condition maximizes the likelihood of detecting the impact of VAC on SSVEP signals and subjective fatigue measures. The 1.0 m distance is considered an intermediate interaction range, representing a comfortable distance for everyday MR interactions (suitable for combined gesture/gaze inputs). The 1.8 m distance represents a typical far-field viewing range for environment-anchored tasks (e.g., spatial anchors, object tracking, or scene layout).

**FIGURE 3:**
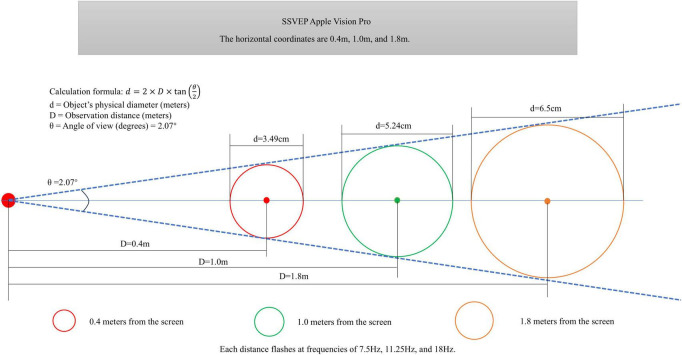
Spatial arrangement of fixed visual angle flash stimulation at different depths.


The flicker stimulus frequencies were 7.5, 11.25, and 18 Hz. The stimuli were green in color and circular in shape (Wu et al., 2025). The spatial layout of fixed visual angle flashing stimuli at varying depths is illustrated in Figure 3. In order to ensure equal retinal input intensity at different depths, all flicker stimuli are designed to maintain a constant visual angle of 2.07°. Consequently, within the virtual 3D space, their actual diameter increases with the specified viewing distance (0.4, 1.0, 1.8 m), as dictated by the geometry of the viewing angle (see Equation 3 and Figure 3). However, during stereoscopic rendering in head-mounted displays, these stimuli naturally appear smaller at greater distances due to perspective projection (Figure 2). This simulates a real-world visual experience while maintaining equivalent demands on the early visual cortex. Each viewing distance corresponded to a specific visual angle; Equation 1 converts this angle to radians, Equation 2 calculates the tangent of half of this angle, and substituting into Equation 3 yields the diameter of each flickering stimulus patch at that distance (resulting diameters: 3.49, 5.24, and 6.5 cm for 0.4, 1.0, and 1.8 m, respectively; see Figure 3). Here, *d* is the physical diameter of the target (in meters), *D* is the viewing distance (in meters), and θ is the visual angle in degrees, specifically 2.07°.

$$\theta_{r\,a\,d}=\theta \times \frac{\pi }{180}=2.07\times \frac{\pi }{180}\approx 0.03613\,r\,a\,d$$ (1)

$$\tan\left(\frac{\theta_{r\,a\,d}}{2}\right)=\tan\left(\frac{0.03613}{2}\right)=\tan\left(0.018065\right)=0.01807$$ (2)

$$d=2\times D\times \tan\left(\frac{\theta }{2}\right)=2\times 1.8\times 0.01807\approx$$

0.06505⁢m=65.05⁢m⁢m (3)

### 2.3 Experimental procedure

A single-target, passive-gaze SSVEP (steady-state visual evoked potential) paradigm was employed to assess signal quality under different conditions. This design enabled the isolation of the effects of distance and environment on the SSVEP response itself.

The presentation of the stimulus: In each trial of the experiment, a single flashed stimulus was presented on the screen. The virtual depth of the stimulus (0.4, 1.0 and 1.8 m) and its flashing frequency (7.5, 11.25, and 18 Hz) were predetermined according to the experimental grouping design.

Participant task: Participants were instructed to passively gaze at the flashing stimulus when it appeared and maintain fixation until it disappeared.

Test structure: At the commencement of each trial, a white fixed cross (“+”) is displayed at the target position for a duration of 1 s, serving as a cue. This is then immediately followed by a single 5-s flash stimulus, after which a 3-s blank screen is presented (rest period). This sequence is repeated 30 times prior to progression to the subsequent distance trial. Within each distance range, one frequency is displayed for 10 flashes, and one distance is displayed for 30 flashes, thus yielding a total of 90 flash trials across three distances. The total duration of the experiment is approximately 13.5 min. Two display scenarios (MR and VR) were tested: Following the completion of one scenario, the participants were permitted a period of rest lasting approximately 10 min before commencing the second scenario. Each scenario was conducted on two occasions (two rounds per condition in MR/VR). Following the administration of the dual-scenario test, participants completed a questionnaire that evaluated ten symptoms of visual fatigue (e.g., dry eyes, double vision) using a 5-point Likert scale (1 = no fatigue, 5 = severe fatigue).

### 2.4 Signal acquisition

**FIGURE 4:**
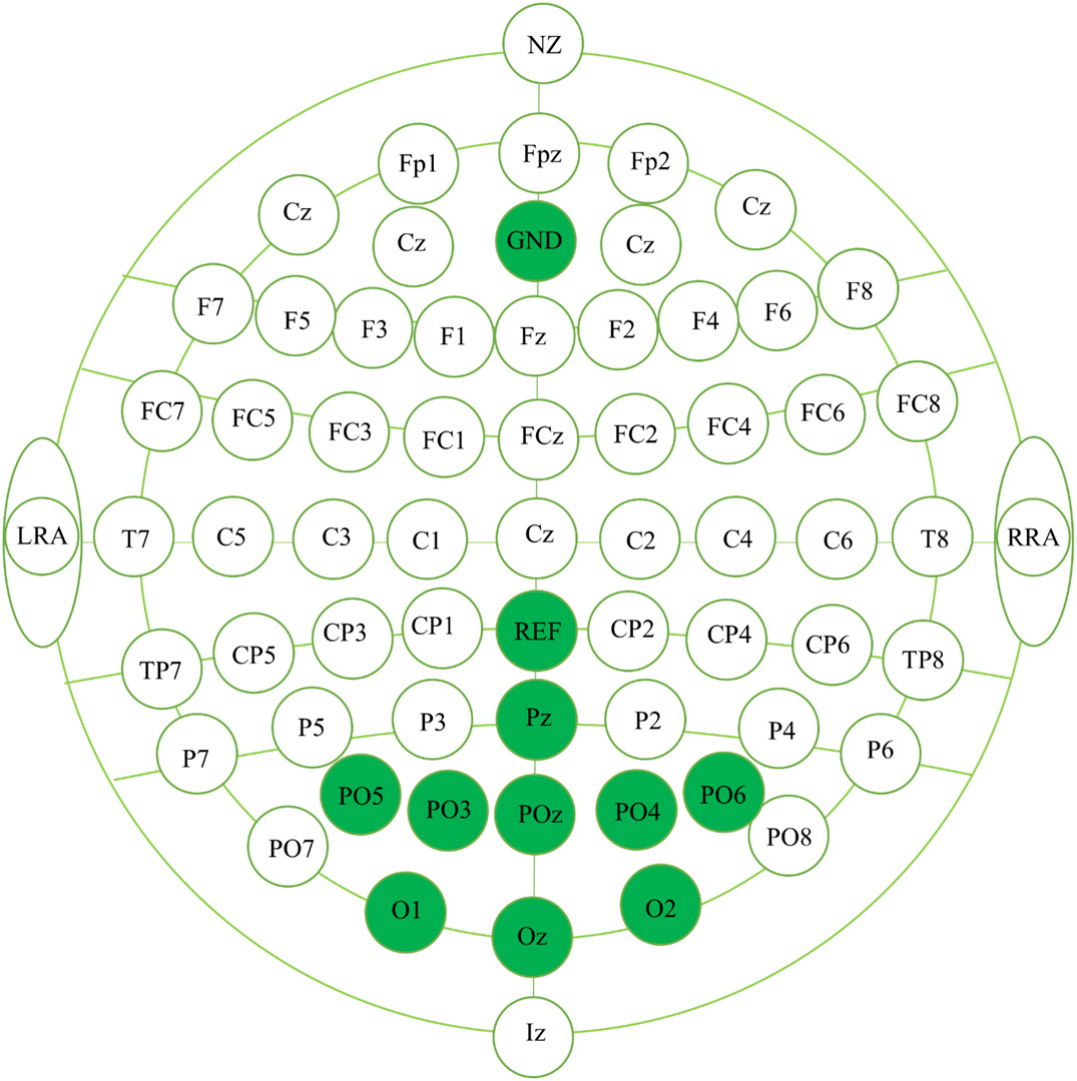
Electrode channel diagram.

Electroencephalography signals were recorded using the Neuracle wearable multimodal EEG cap and NeuroHub software. The EEG electrodes were located at positions Oz, O1, O2, PO3, PO4, PO5, PO6, POz, and Pz, with a ground (GND) and reference (REF) electrode. The electrode channel diagram is shown in Figure 4. All signals were sampled at 1,000 Hz.

## 3 Results

### 3.1 Data processing

Nine EEG channels (Oz, O1, O2, PO3, PO4, PO5, PO6, POz, Pz) were selected for analysis. The continuous EEG data were segmented into trials based on synchronization markers generated by the visual stimulation program. Each SSVEP stimulus period lasted 5 s; thus, the analysis window included a 1-s pre-stimulus baseline and the 5-s stimulus duration (from 1 s before stimulus onset to the end of the 5 s stimulus). EEG data were band-pass filtered from 0.1 to 40 Hz. A spatial filtering technique (such as TRCA) was then applied to enhance the signal-to-noise ratio. The filtered data were down sampled to 250 Hz, and bad segments were removed before further analysis.

### 3.2 Data analysis methods

To comprehensively evaluate the SSVEP characteristics, our quantitative analysis followed a two-pronged approach:

1. Data validation without a training method: This part focuses on assessing the intrinsic quality of the SSVEP signals under different conditions, using metrics such as the Signal-to-Noise Ratio (SNR) and correlation coefficients (from CCA/FBCCA) computed directly on all available trials. The results reflect the fundamental impact of stimulus distance and display environment on the neural response, independent of any classifier calibration.

2. Data validation with a training method: This part evaluates the practical classification performance achievable with the signals. It involves a standard machine learning pipeline, including data preprocessing, a defined procedure for dividing data into training and testing sets, model training, and final accuracy reporting. The classification results (e.g., from TRCA) presented in this study are derived from this process.

The following sections detail the procedures and present the findings accordingly.

#### 3.2.1 Traditional analysis methods (CCA and FBCCA)

All subsequent analyses were implemented in MATLAB R2023b, employing bespoke scripts to invoke the EEGLAB toolbox for the purpose of processing and illustrating the EEG data. NeuroHub is used for raw format conversion and acquisition of electroencephalogram (EEG) data.

First, template matching methods (CCA and FBCCA) requiring no subject-specific model training are employed for classification. For each test trial, its correlation with a predefined set of sine reference templates (corresponding to each stimulus frequency) is calculated. The frequency with the highest correlation is ultimately selected as the classification output.

Two traditional analysis methods, Canonical Correlation Analysis (CCA) and Filter-Bank Canonical Correlation Analysis (FBCCA), were applied to process the EEG data. The essence of CCA is to analyze the overall correlation between two sets of variable (Lin et al., 2006; Bin et al., 2009). In this context, one set is the multi-channel EEG signal *X* = [*x_1_, x*_2_,…., *x_*n*_*], and the other set is an artificially constructed reference signal composed of sine and cosine waves at the stimulus frequency. CCA finds the correlation coefficient between the two resulting waveforms. In practice, CCA seeks linear combinations of the EEG signals and the reference signals such that the correlation between the combined signals is maximized, as shown in Equation 4.


$$\rho \,\left(f_{i}\right)=\frac{E_{\left(x^{T}\,y_{i}\right)}}{\sqrt{E\,\left(x^{T}\,x\right)\,E\,\left(y^{T}\,y\right)}}=\frac{E\,\left(W_{X}^{T}\,X\,Y_{i}\,W_{Y\,i}\right)}{\sqrt{E\,\left(W_{X}^{T}\,X\,X^{T}\,W_{X}\right)\,E\,\left(W_{Y\,i}^{T}\,Y\,Y^{T}\,W_{Y\,i}\right)}}$$ (4)

Specifically, it finds weight vectors *W*_*X*_ and *W*_*Y*_ such that *x = X*^T^*W_*X*_* and *y = Y*^T^*W_*Y*_*, where *Y* represents the reference sine/cosine signals for a given stimulus frequency. Here, *w* and *v* are weighting coefficients, and for each target *i*, the reference signal corresponds to the stimulus frequency *f*_*i*_. The resulting correlation coefficient between *x* and *y* indicates how strongly the EEG corresponds to that stimulus frequency.

The CCA model has been demonstrated to be suitable for scenarios where the frequency of stimulation and the level of noise are both low. It is notable for its low computational complexity and strong real-time performance. However, its limitations stem from its incapacity to accommodate substantial individual variations, consequently yielding low recognition accuracy in intricate backgrounds.

Filter-Bank Canonical Correlation Analysis extends CCA by introducing a filter bank that incorporates both fundamental and harmonic components of the SSVEP (Chen et al., 2015). The original EEG signals are decomposed using Butterworth band-pass filters into multiple sub-band components. For each sub-band component and each stimulus frequency, the correlation coefficient between the EEG sub-band signal and the corresponding reference signals (for all stimuli *Y*_*fk*_, *k* = 1, 2….S, where *k* = 1, 2, …, S, with S being the number of stimuli) is calculated. For the *k*-th stimulus, this yields a vector of correlation coefficients across *N* sub-bands.

These sub-band correlation values are then combined using a weighted sum of squares fusion (Equation 5), where *n* is the sub-band index and Equation 7 is the weight for the *n*-th sub-band defined by Equation 6.


$$\rho_{k}=\left[\begin{array}{c} \rho_{k}^{1}\\ \rho_{k}^{2}\\ \begin{array}{c} \vdots \\ \rho_{k}^{N} \end{array} \end{array}\right]=\left[\begin{array}{c} \rho \,\left(X_{S\,B_{1}}^{T}\,W_{X}\,\left(X_{S\,B_{1}}\,Y_{f_{k}}\right),Y^{T}\,W_{Y}\,\left(X_{S\,B_{1}}\,Y_{f_{k}}\right)\right)\\ \begin{array}{c} \rho \,\left(X_{S\,B_{2}}^{T}\,W_{X}\,\left(X_{S\,B_{2}}\,Y_{f_{k}}\right),Y^{T}\,W_{Y}\,\left(X_{S\,B_{2}}\,Y_{f_{k}}\right)\right)\\ \vdots \\ \rho \,\left(X_{S\,B_{N}}^{T}\,W_{X}\,\left(X_{S\,B_{N}}\,Y_{f_{k}}\right),Y^{T}\,W_{Y}\,\left(X_{S\,B_{N}}\,Y_{f_{k}}\right)\right) \end{array} \end{array}\right]$$ (5)

$${\tilde{\rho }}_{k}=\overset{N}{\underset{\text{n}=1}{\sum }}w\,\left(n\right)•{\left(p_{\text{k}}^{\text{n}}\right)}^{2}$$ (6)

$$w\,\left(n\right)=n^{-1.25}+0.25,n\in \left[1,N\right]$$ (7)

The reference signal frequency corresponding to the maximum value of feature k constitutes the classification result for the EEG signal. A fourth-order, zero-phase Butterworth bandpass filter with a passband of 0.1–40 Hz is employed. This comprises four sub-bands: 0.1–10, 10–20, 20–30, and 30–40 Hz. The four distinct frequency bands are delineated on the basis of the fundamental frequency and harmonic distribution range of SSVEP signals (where the fundamental frequencies of 7.5, 11.25, and 18 Hz, along with their second harmonics, all fall within the 0.1–40 Hz range). This subdivision of the band effectively captures the energy distribution across different frequency components, ensuring that the FBCCA algorithm can efficiently extract stimulus-locked components across each frequency band.

Filter-Bank Canonical Correlation Analysis has been demonstrated to be a suitable approach for scenarios involving frequent stimulation frequencies and significant inter-subject variability in frequency band responses (for instance, in the context of multi-target SSVEP-BCI). However, the utilization of inter-trial temporal correlations is found to be inadequate, resulting in diminished performance metrics when confronted with substantial interference.

#### 3.2.2 Spatial filtering enhancement

To further improve the separability of SSVEP features, task-related component analysis (TRCA) was employed for spatial filtering. Task-Related Component Analysis (TRCA) is a spatial filtering method for extracting stable task components from multi-channel EEG signals. Its core principle involves identifying an optimal spatial filter that maximizes signal consistency across multiple task repetition (Nakanishi et al., 2018).

Given *K* training samples{*X_1_,X_2_,……X_*k*_* under a specific task category (where each *X*_*k*_∈ *R*^*C* × *T*^, *C* denotes the number of channels, and *T* denotes the number of time points), TRCA aims to solve the following generalized eigenvalue problem to obtain the optimal spatial filter *w*, as shown in Equation 8.


$$S^{\left(t\right)}\,w=\lambda \,S^{\left(\text{n}\right)}\,w$$ (8)

Where: *S*^(^*^t^*^)^ is the inter-trial covariance matrix, and maximizing this objective maximizes the consistency of signals across trials. *S*^(^*^n^*^)^ is the normalization constraint matrix, typically set to the overall covariance matrix of all trials or the identity matrix *I*. The formula for the spatial filter weight matrix is *W* = *R^C^* × *^k^*, where each column *W*_*k*_ corresponds to an optimal filter for a target frequency, used to enhance target SSVEPs and suppress noise.

In practice, the filter set *W* = [*w_1_,……w_*N*_*] is often constructed using the eigenvectors corresponding to the first *N* largest eigenvalues, serving to extract multidimensional features.

Task-related component analysis has been demonstrated to exhibit significant advantages in short-time windows and multi-target scenarios; however, its reliance on sufficient individual training data is a key drawback.

The signal-to-noise ratio (SNR) was utilized to assess the quality of steady-state visual evoked potential (SSVEP) signals, as this metric effectively reflects the intensity of signal energy at the target frequency relative to background noise. For each trial, the power spectral density of the EEG signal during the 5-s stimulus presentation period was calculated. SNR is defined as the ratio of the sum of power at the target frequency (*F0*) and its second harmonic (*2F0*) to the average background noise power in adjacent frequency bands (typically excluding *F0* ± 0.1Hz and *2F0* ± 0.1 Hz). This is expressed in decibels. The specific calculation is given by Equation 9.


$$S\,N\,R\,\left(d\,B\right)=10\times \log_{10}\left(\frac{P_{f_{0}}+P_{2\,f_{0}}}{P_{n\,o\,i\,s\,e}}\right)$$ (9)

In this context, *P*_*f0*_ and *P*_2f0_ represent the power at the target frequency *f*_0_ and the second harmonic frequency 2*f*_0_, respectively, while *P*_*noise*_ denotes the average power of background noise.

For all analyses involving model training and classification performance evaluation, a rigorous data partitioning strategy was adopted. The classification task aimed to distinguish three flicker frequencies (7.5, 11.25, and 18 Hz) under a given display environment and specific stimulus distance. Therefore, for each scenario, we constructed a dataset comprising 90 trials (3 distances × 3 frequencies × 10 trials per frequency).

This combined dataset (90 trials per condition) was subjected to 10-fold cross-validation: within each fold, trials were randomly allocated such that 70% (63 trials) formed the training set (used to calibrate algorithm parameters, e.g., computing TRCA spatial filters); the remaining 30% (27 trials) constituted an independent test set.

**FIGURE 5:**
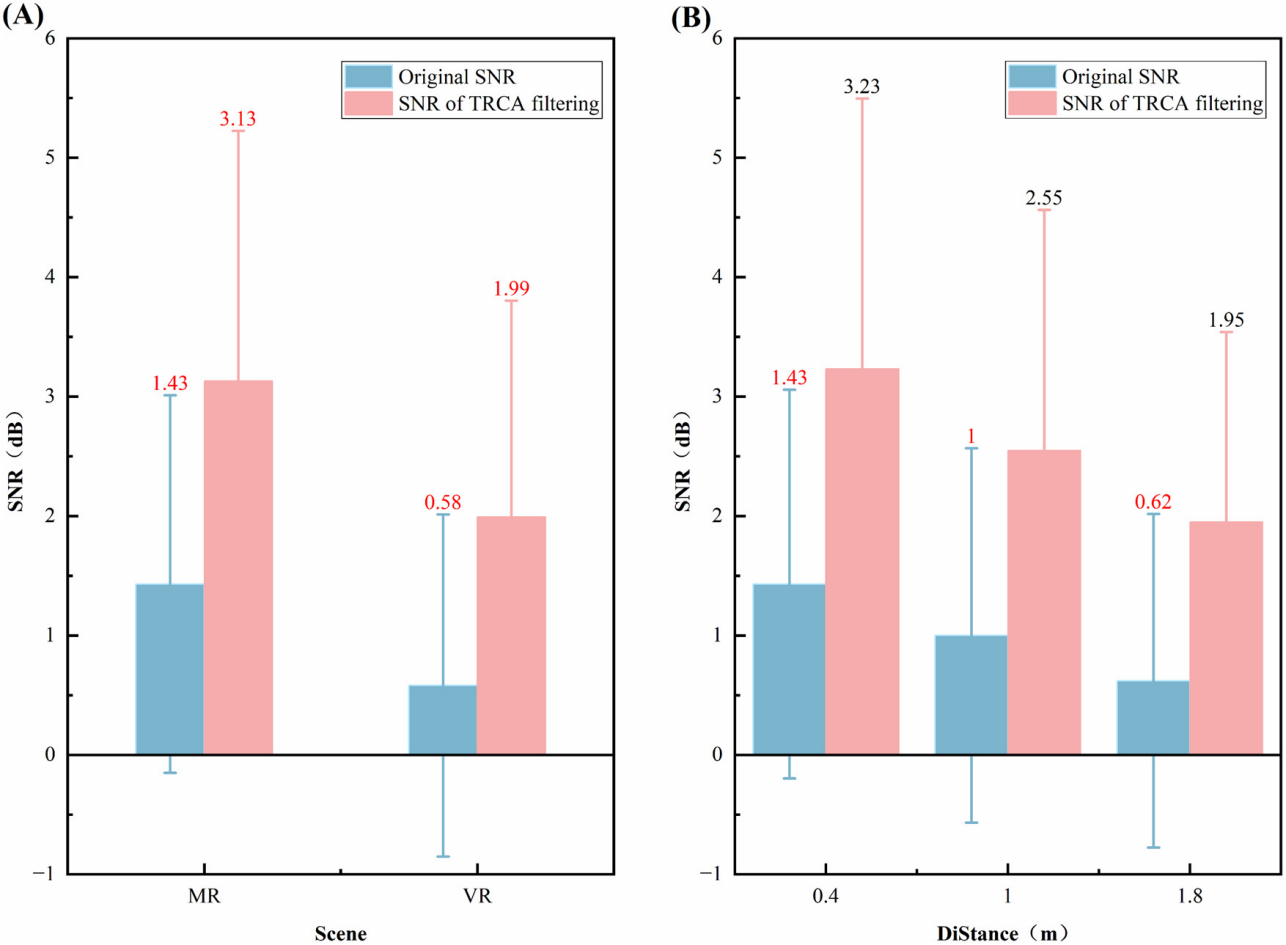
Comparison of spatial filtering effects at different distances and scene. **(A)** Effects of spatial filtering in different scenario. **(B)** Effects of spatial filtering in different distances.

The effects of spatial filtering are compared across different scenarios and distances in Figures 5A, B, which contain two subfigures. Figure 5A illustrates the effects of spatial filtering under different scenarios, while Figure 5B shows the effects at varying distances. Initially, the SNR of the original signal is represented by the blue bars, which demonstrate an SNR of 1.43 dB in the MR environment and 0.58 dB in the VR environment. The pink bars represent the signal SNR after TRCA spatial filtering, indicating a substantial enhancement. In the MR environment, the SNR increased to 3.13 dB; in the VR environment, it increased to 1.99 dB. As illustrated in Figure 5B, the signal-to-noise ratio (SNR) is demonstrated at varying distances for the subjects. The initial SNR at distances of 0.4, 1.0, and 1.8 m was recorded at 1.43, 1, and 0.62 dB, respectively. Following the implementation of TRCA filtering, the SNR demonstrated enhancement, reaching 3.23, 2.55, and 1.95 dB, respectively.

Overall, these results (Figure 5) reveal that SSVEP signal quality in the MR environment (both before and after filtering) was better than in the VR environment, and that SSVEP signal quality decreased as viewing distance increased (both before and after filtering). The effectiveness of the trained TRCA filters was validated by comparing the Signal-to-Noise Ratio (SNR) before and after filtering (Figure 5). The significant SNR increase across all scenarios and distances demonstrates the successful training and generalization capability of the TRCA spatial filters.

In this study, a square-wave modulation technique was used to drive the visual flicker stimuli, generating a high-contrast rectangular flicker waveform by controlling the light source on/off timing with a digital square-wave signal (Vialatte et al., 2010). In essence, the flicker is governed by a signal *S*(*t*) that follows a defined temporal rule specifying when the stimulus is “on” and “off,” with its mathematical expression given by Equation 10.


$$S\,\left(t\right)=\left\{ \begin{array}{c} I_{\max}\\ 0 \end{array}\right.\,\begin{array}{c} i\,f\,0\leq t\,m\,o\,d\,T<\alpha \,T\\ o\,t\,h\,e\,r \end{array}\}$$ (10)

Key parameters of this flicker signal include:

① Period (*T*): The period is directly related to the flicker frequency f, satisfying *T* = 1/*f*. For example, if the flicker frequency *f* = 10 Hz (10 flashes per second), then the period *T* = 0.1 s (one “on-off” cycle every 0.1 s).

② Duty cycle (α): In this study, α = 0.5 (fixed), meaning the “on” duration and “off” duration are equal. Specifically, the stimulus is on for *αT* and off for (1−α) *T* each cycle. With α = 0.5, the on and off times are each half of the period (Notbohm et al., 2016).

③ Light intensity output: Defined as 1 at maximum brightness (when the stimulus is on) and 0 with no light output (when the stimulus is off).

Hardware implementation (digital signal to actual flicker): Presenting the flicker stimulus requires coordination between software generation and hardware rendering, with parameter adaptation to ensure the waveform is displayed in full: (1) Signal generation: Unity software generates a square-wave sequence following the above *S*(*t*) rule (i.e., the timing instructions for on/off). (2) Rendering output: These instructions are rendered on the Apple Vision Pro, which has a fixed refresh rate of 90 Hz (90 frames per second). A frame-multiplicity adaptation formula is applied: to avoid the flicker waveform being “cut off” by the device’s refresh, the flicker frequency *f* must satisfy *f* = 90/n (where n is a positive integer). For example, if *n* = 8, then *f* = 90/8 = 11.25 Hz, corresponding to eight frames per flicker cycle (with four frames on and four frames off, given α = 0.5). This exactly matches the device’s frame rate, ensuring the full flicker waveform is rendered without distortion (Nakanishi et al., 2015).

### 3.3 Effect of stimulation distance on accuracy

Accuracy calculation: The classification accuracy under specific conditions (e.g., at a distance of 0.4 m) is calculated as shown in Equation 11:


$$A\,c\,c\,u\,r\,a\,c\,y=\frac{N_{c\,o\,r\,r\,e\,c\,t}}{N_{t\,o\,t\,a\,l}}\times 100\%$$ (11)

*N*_*total*_ denotes the total number of trials under a specific condition. *N*_*correct*_ denotes the number of correctly classified trials (i.e., the analyzed frequency matches the actual stimulus frequency). In the event of the analyzed frequency matching the actual stimulus frequency presented during the trial, the trial is deemed to have been correctly completed. This method directly assesses the intensity and specificity of neural responses elicited by each stimulus condition. The present study is applicable to a comparative analysis, the objective of which is to evaluate the inherent distinguishability of SSVEPs across varying depths and display modes. The final accuracy rate is reported as the mean ± standard deviation.

**FIGURE 6:**
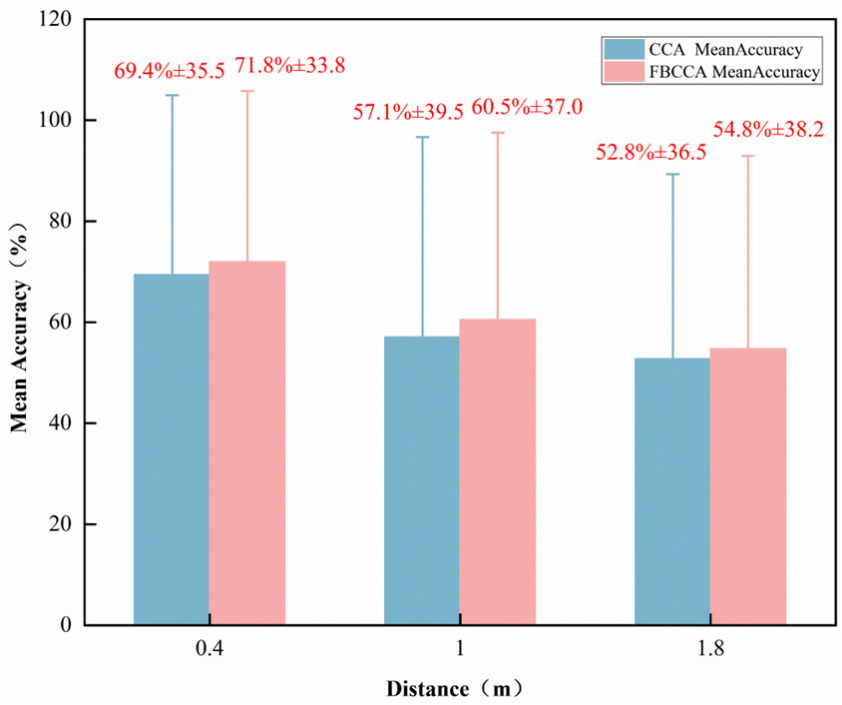
Classification accuracy of steady-state visually evoked potentials (SSVEP) for 10 subjects under three different stimulus distances (time window ranging from 0 to 5 s).

As demonstrated in Figure 6, the classification accuracy of SSVEP is shown to vary according to the different stimulus distances (time window from 0 to 5 s). The horizontal axis denotes varying distances (0.4, 1.0, 1.8 m), whilst the vertical axis indicates accuracy. The blue bars represent the CCA classification accuracy at varying distances, while the pink bars denote the FBCCA classification accuracy at different distances. As the stimulus distance increased, the classification accuracy gradually decreased for all participants. Across both classification algorithms tested, FBCCA generally yielded better accuracy than CCA, and the effect of stimulus distance on performance was evident. In both the CCA and FBCCA results, the accuracy was highest at the 0.4 m distance (CCA: 69.4% ± 35.5; FBCCA: 71.8% ± 33.8), followed by 1.0 m (CCA: 57.1% ± 39.5; FBCCA: 60.5% ± 37.0), and lowest at 1.8 m (CCA: 52.8% ± 36.5; FBCCA: 54.8% ± 38.2). FBCCA significantly enhances the representation capability of SSVEP signals through filter bank decomposition, multi-subband fusion, harmonic utilization, and weighted optimization, while preserving the computational efficiency of CCA. Consequently, FBCCA outperforms traditional CCA in terms of classification accuracy and noise robustness.

### 3.4 Relationship between CCA correlation coefficient and SNR relationship between FBCCA and SNR

**FIGURE 7:**
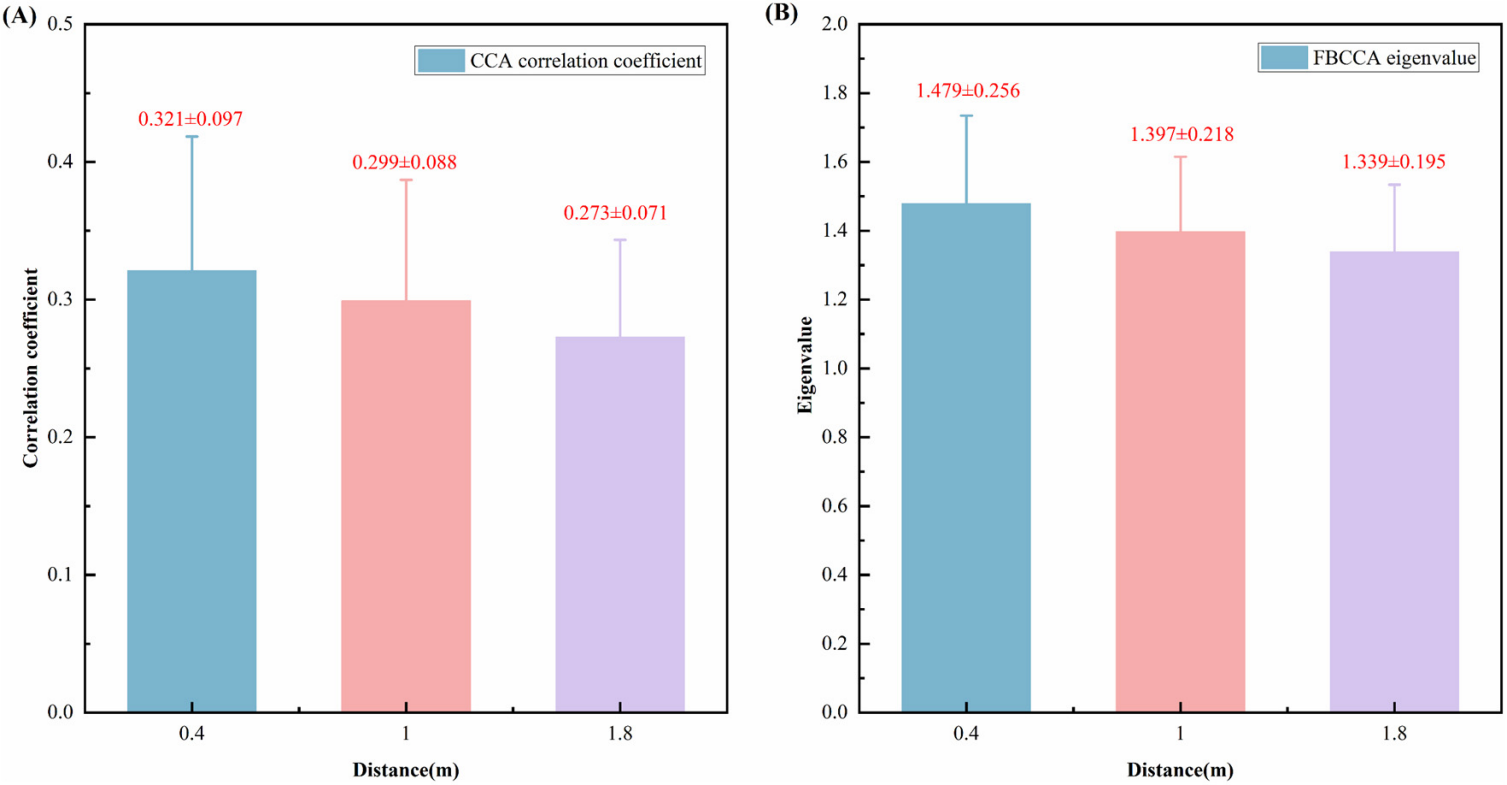
CCA correlation coefficients at different distances and FBCCA eigenvalues. **(A)** CCA correlation coefficients at different distance. **(B)** FBCCA eigenvalues at different distances.

As demonstrated in Figure 7, the CCA correlation coefficients and FBCCA eigenvalues are observed to vary with respect to the distance from the subject to the stimulus. Figure 7A presents typical correlation coefficients from the canonical correlation analysis (CCA) conducted at three stimulus distances (0.4, 1.0, and 1.8 m). The quantification of the linear relationship between multi-channel EEG signals and the reference sinusoidal template at the target flicker frequency is achieved by the coefficients. The data are presented as the mean ± standard deviation. The mean CCA correlation coefficient is observed to be at its highest at a distance of 0.4 m (0.321 ± 0.097), followed by 1.0 m with a mean CCA correlation coefficient of 0.299 ± 0.088, and finally 1.8 m with a mean CCA correlation coefficient of 0.273 ± 0.071. As the distance of the stimulus increases, the CCA correlation demonstrates a decreasing trend.

As illustrated in Figure 7B, the Filter-Bank Canonical Correlation Analysis (FBCCA) eigenvalues were obtained at three distinct stimulus distances. The aforementioned eigenvalues are indicative of the strength of the optimized features that have been extracted via the FBCCA algorithm. The data are presented as the mean ± standard deviation. In accordance with the trend observed in (A), the FBCCA eigenvalues demonstrate a decline in value as the stimulus distance is increased. The blue bars in Figure 7 represent the CCA correlation coefficient and FBCCA eigenvalues at a distance of 0.4 m, the pink bars represent those at 1.0 m, and the purple bars represent those at 1.8 m.

**FIGURE 8:**
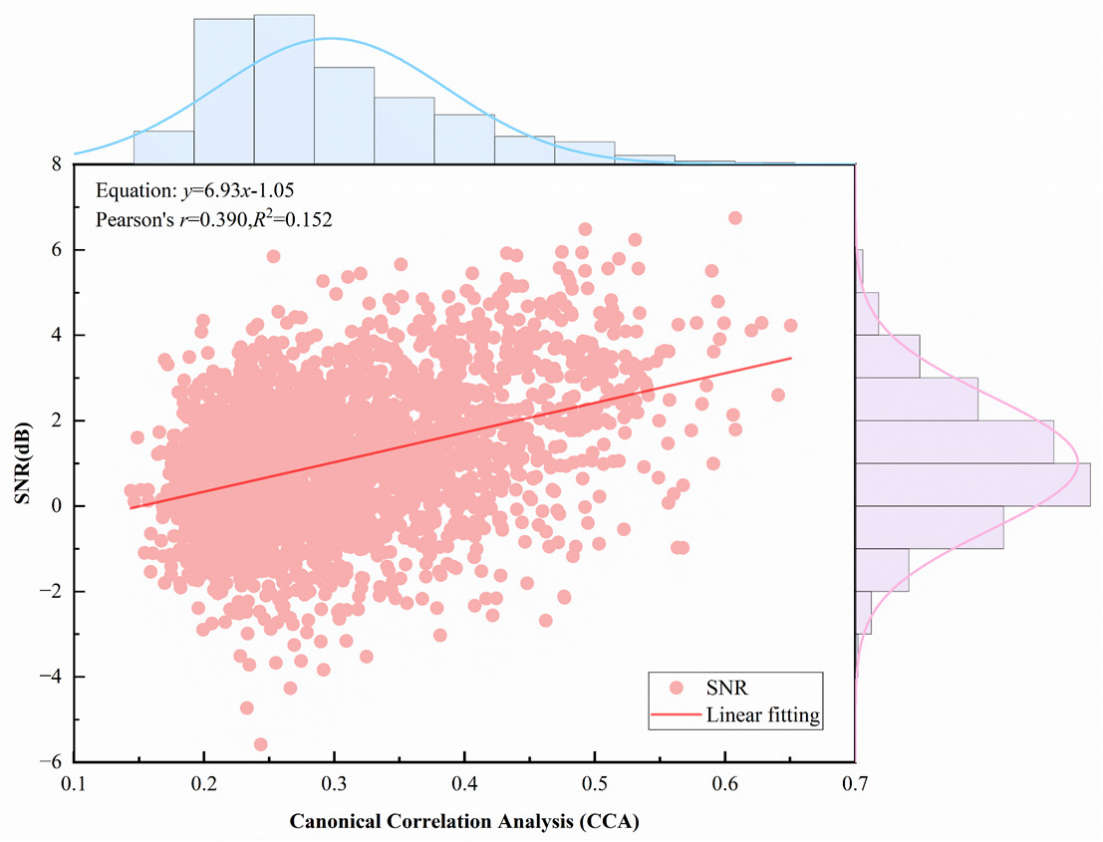
Canonical correlation analysis (CCA) correlation coefficient and signal-to-noise ratio (SNR) relationship.

**FIGURE 9:**
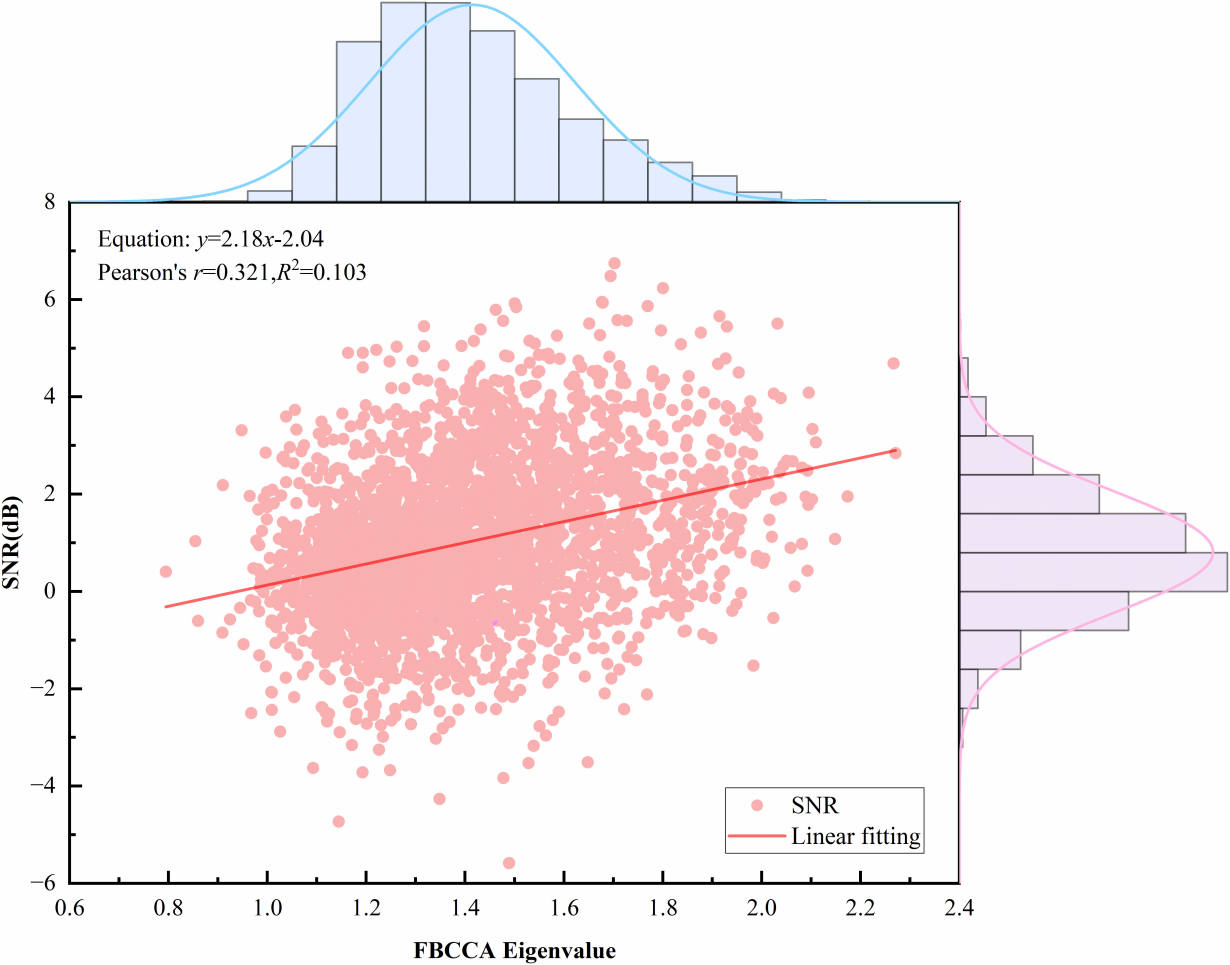
Relationship between Filter-Bank Canonical Correlation Analysis (FBCCA) eigenvalues and signal-to-noise ratio (SNR).

Figure 8 illustrates the relationship between the CCA correlation coefficient and SNR. A linear fit was performed to relate the “theoretical signal (*x*)” to the “actual observed signal (*y*),” yielding the equation *y* = 6.93x–1.05. The slope *a* = 6.93 (*a* > 0) indicates that the CCA correlation coefficient is proportional to SNR. Pearson’s correlation *r* = 0.390 (*r* > 0) further confirms a positive correlation: as the theoretical signal strength increases, the observed signal strength also increases. Similarly, Figure 9 shows the relationship between FBCCA eigenvalues and SNR, which is also proportional.

### 3.5 Subjective visual fatigue evaluation

After the experiment, participants completed a visual fatigue questionnaire with 10 items, each rated on a five-point scale (1 = no fatigue, 5 = severe fatigue). The statistical analysis software used was SPSS, employing the Wilcoxon signed-rank test to compare differences in subjective symptom scores between the MR and VR environments. In view of the fact that ten comparisons were performed, the Bonferroni correction was applied in order to control Type I errors, with the significance level set at α = 0.005.

The results of the Wilcoxon signed-rank test are displayed in Table 1. In the case of binocular diplopia, the scores in the VR environment (median = 3.0, interquartile range = 2.0–4.0) were higher than those in the MR environment (median = 2.0, interquartile range = 1.0–3.0), with a Z statistic of -2.121 and a *p*-value of 0.034. The findings of this study indicate that discomfort is exacerbated in the VR environment in comparison to the MR environment. However, subsequent to the implementation of the Bonferroni multiple comparison correction (corrected α = 0.005), this discrepancy did not attain statistical significance (*p* > 0.005). Despite the absence of statistical significance, a consistent tendency was observed across symptoms, including diplopia, difficulty focusing, and dry eyes, where VR-environment scores tended to be higher. The remaining nine symptoms demonstrated no statistically significant differences in score between the two environments (all *p* > 0.005). It is noteworthy that a tendency towards elevated VR scores in comparison to MR was evident for eight symptoms (negative Z-scores), particularly in the cases of binocular diplopia (five participants VR > MR, zero participants MR > VR) and difficulty in fixation (six participants VR > MR, one participant MR > VR).

**TABLE 1:** The following study will compare subjective visual symptom scores in mixed reality and virtual reality environments for headaches (Wilcoxon signed-rank test).

Symptom	MR score (median, interquartile range)	VR score (median, interquartile range)	Negative-rank number (VR < MR)	Positive-order number (VR < MR)	Number of bound values	*Z*-value	*P*-value
Eyes feel sore	2.0 (2.0, 2.0)	2.0 (1.75, 3.25)	1	3	6	−1.134	0.257
Dry eyes	2.0 (1.75, 3.25)	2.5 (2.0, 3.75)	0	3	7	−1.732	0.083
Blurred vision	2.5 (2.0, 3.0)	2.5 (2.0, 3.0)	3	3	4	−0.333	0.739
Double vision	2.0 (1.0, 3.0)	3.0 (2.0, 3.75)	0	5	5	−2.121	0.034
Difficulty focusing	2.0 (2.0, 3.0)	2.5 (2.0, 3.75)	1	6	3	−1.890	0.059
Eye pain or stinging sensation	1.0 (1.0, 1.0)	1.0 (1.0, 1.0)	0	2	8	−1.414	0.157
Vertigo	1.0 (1.0, 1.0)	1.0 (1.0, 1.0)	0	1	9	−1.000	0.317
Visual fatigue	2.5 (2.0, 3.0)	3.0 (2.75, 4.0)	1	4	5	−1.342	0.180
Declining attention	3.0 (2.0, 3.0)	3.0 (2.0, 3.0)	2	2	6	−0.378	0.705
Headache	1.0 (1.0, 1.0)	1.0 (1.0, 1.0)	0	1	9	−1.000	0.317

The interquartile range (IQR) is reported as the 25th and 75th percentiles. Data are described as median (IQR). The Wilcoxon signed-rank test was used to compare the mixed reality (MR) and virtual reality (VR) environments. Since 10 comparisons were performed, Bonferroni multiple comparison correction was applied, setting the corrected significance level at α = 0.005. A negative Z-score indicates a higher VR score than an MR score (based on negative ranks).

## 4 Discussion

### 4.1 Stimulus distance decay effect

It is evident from the findings that both the FBCCA and CCA algorithms demonstrate a marked decline in accuracy as the distance of the stimulus increases. The TRCA algorithm also demonstrates that SNR decreases progressively with increasing stimulus distance, whether measured before or after filtering. The findings from both TRCA experiments have shown that TRCA spatial filtering enhances the signal-to-noise ratio (SNR) of SSVEP signals under all experimental conditions. This finding is in alignment with the seminal work of Nakanishi et al. (2018), which provides robust confirmation of the universal effectiveness and robustness of the TRCA algorithm as a powerful feature extraction tool for improving SSVEP-BCI performance. The fundamental mechanism of this system is to enhance consistency across trials, thereby enabling effective extraction of task-related neural responses from multi-channel EEG signals. This process involves the suppression of task-irrelevant background noise and artifacts.

As the distance increases, the average SNR decreases. This phenomenon can be attributed to the induction of an accommodation-vergence conflict (VAC) by MR headsets, which results in visual fatigue over protracted period (Hoffman et al., 2008). In fixed-focus HMDs, the presentation of a “visually distant” virtual target has been demonstrated to trigger VAC. This conflict has been shown to cause subjective discomfort and to disrupt the stability of visual signal processing at the neural level, manifesting as reduced SSVEP signal synchrony under long-distance conditions. Specifically, VAC has been demonstrated to induce instability within binocular coordination and focusing mechanisms, consequently impairing the visual cortex’s capacity to respond synchronously to flicker stimuli.

As the distance increases, the average signal-to-noise ratio (SNR) exhibits a downward trend. This phenomenon directly corroborates the findings of Zhou et al. (2021), who demonstrated that visual evoked potentials (VEPs) represent a core limiting factor for head-mounted display (HMD)-based visual interfaces, as they disrupt binocular coordination and diminish the synchrony of visual evoked potential signals.

### 4.2 Frequency advantage effect

**FIGURE 10:**
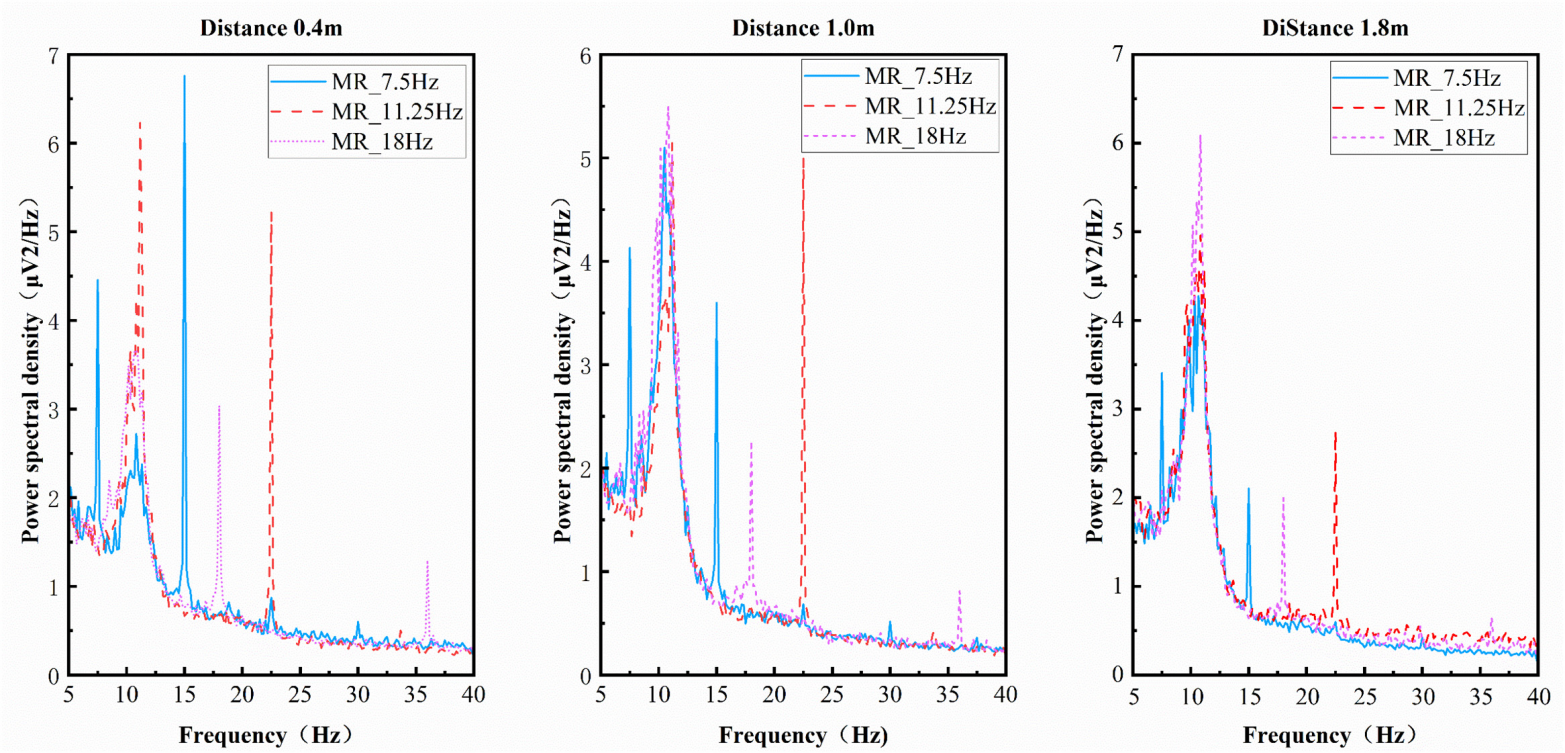
Amplitude spectra at different frequencies for mixed reality (MR) scenes at varying distances.

**FIGURE 11:**
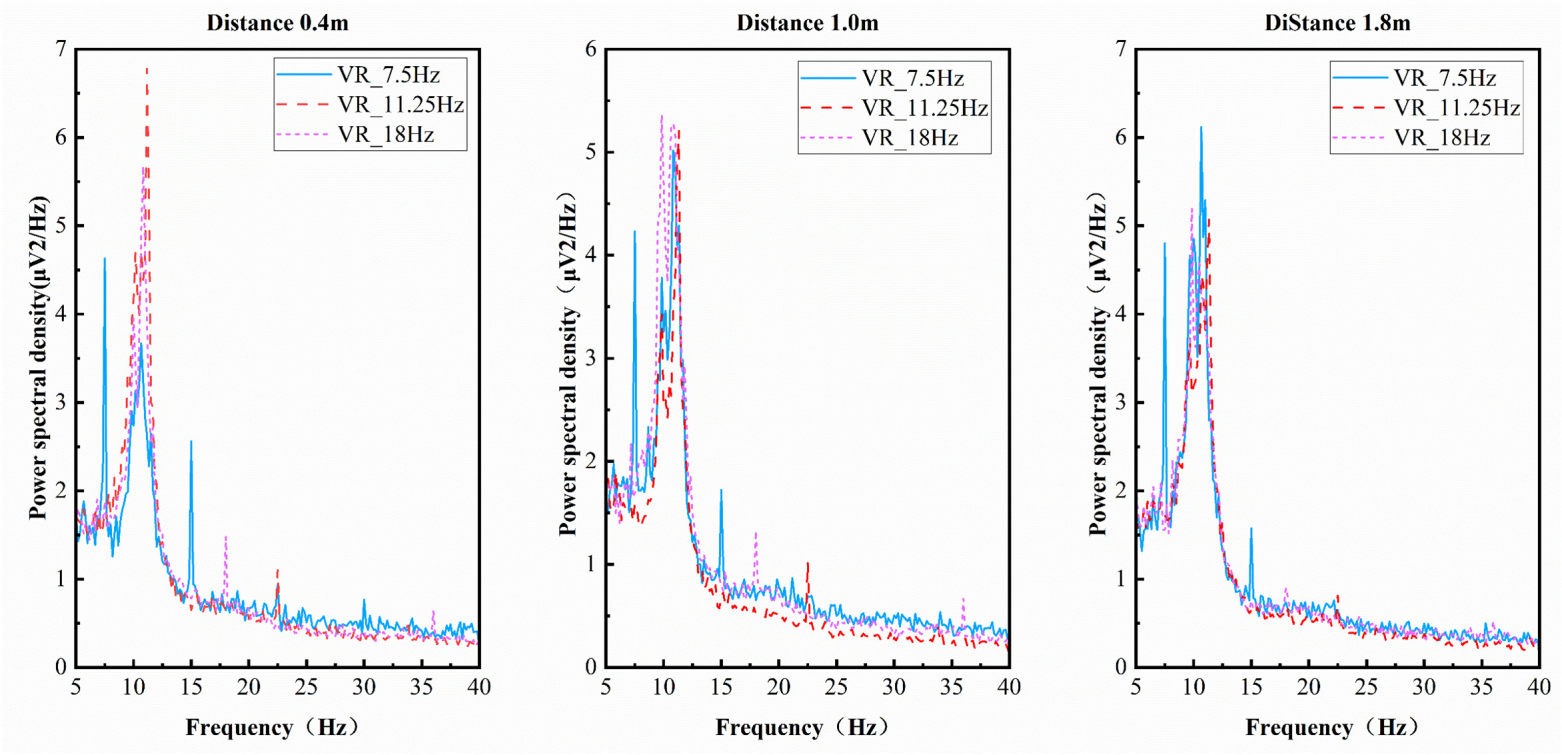
Amplitude spectra at different frequencies across varying distances within the virtual reality (VR) scene.

The quality of SSVEP signals was compared at different distances across a range of scenarios by means of analysis of amplitude and SNR. Figure 10 shows the average amplitude spectra across different frequencies at various distances in the MR scenario, while Figure 11 displays the average amplitude spectra across different frequencies at various distances in the VR scenario. It was demonstrated that stimuli presented at each distance induced both fundamental frequencies and harmonics at corresponding frequencies in both the MR and VR scenarios (Vialatte et al., 2010). This finding indicates that they generated corresponding visual evoked potentials and brain responses to visual stimuli were achieved. The figures reveal that the amplitude at 11.25 Hz is higher than that at 7.5 and 18 Hz in both the MR and VR scenarios.

The data indicates that in both MR and VR scenarios, the amplitude at 11.25 Hz exceeds that at 7.5 and 18 Hz, aligning with the focus of Liu et al. (2024) on exploring stereoscopic vision’s impact on SSVEP. The research demonstrates that the introduction of 3D stimuli in VR enhances the user experience and achieves optimal performance in the mid-frequency range. In the context of the VR scenario, the mid-frequency 11.25 Hz-which falls within the optimal response band (10–15 Hz) of the visual system-also yielded the highest signal-to-noise ratio. This finding is consistent with the observation that “mid-frequency stimuli may be more adept at integrating 3D spatial cues.” However, the VR accuracy exhibited by the present study was found to be lower than that reported by others, with a maximum of 54.8% ± 38.2% at 0.4 m. This disparity can be attributed primarily to the fundamental differences in experimental paradigms employed in this study. In contrast, Liu et al. (2024) employed deeply fixed static 3D stimuli with an active gaze-selection task. By contrast, the present study sought to systematically quantify the impact of depth variation (0.4–1.8 m) using a passive gaze-following paradigm to isolate the VAC effect. The latter approach may have reduced neural responses modulated by active attention. It is noteworthy that the two studies, taken together, indicate that mid-frequency stimulation (approximately 11 Hz) in combination with 3D visual spatial characteristics functions as an effective parameter for optimizing SSVEP response quality, thus providing a valuable reference point for stimulus parameter selection in immersive BCI. This establishes unified parameter guidelines for immersive BCI design.

**FIGURE 12:**
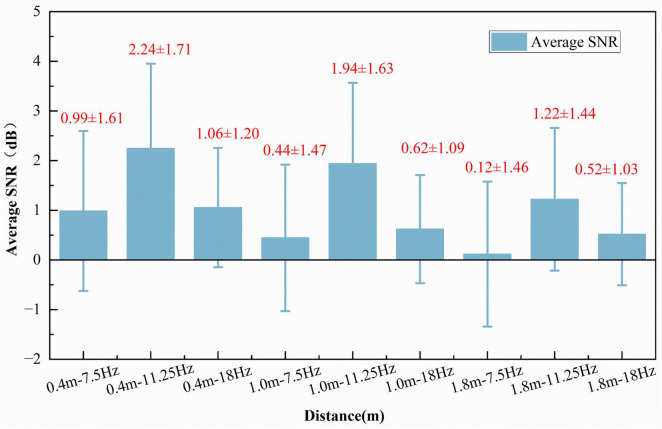
Average signal-to-noise ratio (SNR) at different distances and frequencies.

As illustrated in Figure 12, the mean SNR outcomes are demonstrated at varying distances and frequencies. The average SNR varies across different distances and frequencies, with the highest average SNR observed at 0.4 m–11.25 Hz. The results indicate that the SSVEP amplitude and signal-to-noise ratio induced by the 11.25 Hz stimulus are the highest. Although 7.5, 11.25, and 18 Hz all satisfy the integer sub-division constraint relative to the display refresh rate (90 Hz), ensuring temporal precision, the surface advantage of 11.25 Hz lies in its proximity to the physiologically optimal response band of the visual system (∼10–15 Hz). This phenomenon is of particular interest as it overlaps with the upper edge of the Alpha band in the visual cortex, thereby enabling high levels of neural efficiency and inducing lower levels of visual fatigue.

### 4.3 Differential effects across different scenarios

In the context of MR and VR scenarios, MR scenes have been observed to exhibit a higher SNR in comparison to VR scenes. The following factors have been identified as contributing to this outcome: (1). It is evident that MR scenes consist of physically real objects, whereas VR scenes comprise virtually rendered graphics. The phenomenon of flicker in the context of virtual reality (VR) is influenced by factors such as rendering frame rate stability and vertical synchronization. (2). In the majority of cases, the user’s field of view in MR environments remains static, with continuous lighting that lacks the “global refresh noise” that is inherent in digital displays. The superior quality of SSVEP signals in MR environments can be attributed to two key factors. Firstly, the stable visual background provided by the real world, and secondly, the lower systemic visual noise. Conversely, pure VR environments rely on globally refreshed pixelated displays, which potentially introduce greater visual noise (Maimone and Wang, 2020). Zhou et al. (2021) concentrated on augmented reality (AR) displays, observing that the mitigation of visual-spatial conflict is contingent on the utilization of natural depth cues. The present findings provide further confirmation that mixed reality (MR) provides such cues by integrating real-world backgrounds, whereas VR’s fully virtual environments eliminate these cues, leading to more severe visual-spatial conflicts.

The visual cortex of the brain has been observed to demonstrate a more uniform neural response to stable backgrounds, thereby establishing a low-noise baseline. In the context of VR scenarios, the entirety of the field of view is displayed via pixelated screens, thereby introducing global screen refresh noise. Virtual environments have the capacity to incorporate dynamic textures and light/shadow changes, thereby introducing additional visual noise that increases non-SSVEP-related brain activity (e.g., motion-evoked visual potentials). This, in turn, has the potential to obscure target signals. (3). In MR scenarios, the eyes of the user receive natural light, and the accommodation-convergence response aligns more closely with the user’s physiological habits, resulting in reduced visual fatigue. The closed display of VR systems forces the eyes to focus at a fixed distance, which can easily induce visual fatigue (VIMS). Increases in fatigue levels have been shown to result in an increase in physiological artifacts, such as blinking and eye movements. This, in turn, has the effect of contaminating electroencephalogram (EEG) signals with non-target noise.

The dual advantages of MR environments in signal stability and user comfort stem primarily from their “virtual-real fusion” characteristics: they provide a stable visual reference system approximating the real world to reduce neural noise, while preserving partial natural visual cues to mitigate VAC and associated fatigue. This provides robust empirical evidence in support of the selection of MR as the optimal display platform for SSVEP-BCI in applications necessitating extended use and optimal comfort, such as neural rehabilitation.

### 4.4 Limitations and future prospects

Despite the valuable insights provided by this study for the design of SSVEP-based BCIs in MR/VR environments, there are several limitations that require exploration in future work.

Firstly, all participants in this study were young adults with normal vision (mean age 23 ± 3 years). The study of visual accommodation ability is significantly influenced by age. As individuals age, there is a decline in lens elasticity and a weakening of accommodation capacity. This age-related decline in visual accommodation and convergence function clinically manifests as reduced accommodation range and receded convergence point, a phenomenon clearly demonstrated in individuals with early presbyopia (Alrasheed and Aljohani, 2024). Consequently, the signal attenuation and visual fatigue observed in this study due to accommodation-vergence conflict (VAC) at different distances may be more pronounced in elderly populations. The transferability to more extensive age demographics, notably elderly individuals necessitating assistive technologies, may be constrained. It is recommended that future research recruit participants across diverse age cohorts in order to systematically evaluate how age modulates VAC effects on SSVEP-BCI performance. This would enable the development of more inclusive and adaptive interaction solutions.

Secondly, while the mixed reality environment in the experiment was based on real-world scenarios, it did not strictly control environmental visual complexity (such as background textures, lighting variations, and dynamic distractions). The present study primarily focused on the fundamental display differences between “MR vs. VR” (blending real and virtual elements vs. fully virtual environments) and identified MR’s advantages in signal stability and subjective comfort. However, it should be noted that real-world MR application scenarios vary significantly. A visually cluttered, unevenly lit MR environment may introduce background “noise” far exceeding the relatively tidy laboratory setup, potentially weakening or even reversing the signal quality advantages observed for MR over VR in this study. It is imperative that future research efforts involve the replication of this experiment within systematically controlled environments of varying complexity. This will serve to elucidate the specific relationship between environmental characteristics and SSVEP signal robustness. The outcome of this research will provide more refined guidance for environmental design in practical applications.

Thirdly, the assessment of VAC-induced fatigue in this study was primarily reliant upon subjective questionnaires and synchronous signal metrics. In order to obtain more objective and longitudinally comparable measures of visual system fatigue, subsequent research should incorporate pre- and post-exposure SSVEP amplitude measurements under controlled conditions. Monitoring alterations in this physiological biomarker over time would enable direct quantification of performance degradation, thereby providing stronger evidence of the impact of VAC and environmental factors.

Notwithstanding these limitations, the core finding-that stimulus distance, display environment, and stimulus frequency interactively influence SSVEP signal quality-provides clear empirical support for optimizing immersive BCI systems.

## 5 Conclusion

In this study, we investigated three stimulus distances using an Apple Vision Pro headset and compared MR and VR display scenarios. Different stimulus distances yielded distinct classification results, and the combination of distance and display environment produced differences in performance. Classification results across distances were influenced by factors such as stimulus brightness and the vergence-accommodation conflict, while differences between MR and VR scenarios were influenced by the stability of the stimulus source (physical vs. virtual), background noise suppression, visual fatigue, and artifact control. Among the three tested distances (0.4, 1.0, 1.8 m) in the MR headset, the 0.4 m flickering stimulus achieved the highest SNR. Comparing MR with VR, the MR scenario yielded better SNR. These core findings carry substantial implications for the design and clinical translation of brain-computer interfaces (BCI) and neural prostheses based on steady-state visual evoked potentials (SSVEP). Visual fatigue and discomfort induced by VAC have long been core obstacles to achieving long-term user compliance and clinical adoption of immersive BCI systems. MR’s superior performance in alleviating VAC and reducing fatigue directly enhances the practicality of SSVEP-BCI in prolonged usage scenarios, such as rehabilitation training. Differences in visual fatigue between MR and VR are likely related to the VAC mechanism: in the VR scenario, the conflict between fixed focal distance and virtual depth led to pronounced diplopia (*p* = 0.034), supporting the VAC theory; the MR scenario preserved natural visual cues (such as real depth), resulting in milder diplopia symptoms (effect size *d* = 0.858), consistent with the SNR advantage observed (see section “3.5 Subjective visual fatigue evaluation”).

This study employed a square-wave modulation technique to achieve a high-contrast rectangular flicker waveform, providing a reliable stimulus generation method for investigating SSVEP characteristics at different depths. In future work, we can further focus on dynamic optimization of the duty cycle parameter to specifically alleviate visual fatigue for near-distance stimuli. The present study employed square wave modulation with a fixed duty cycle (α = 0.5) to drive visual stimuli. While the device was able to generate stable flicker waveforms, the sustained alternation between illumination and darkness may intensify accommodation-vergence conflict (VAC) in close-range scenarios (e.g., 0.4 m), potentially exacerbating ocular accommodation strain and visual discomfort. Subsequent research could tailor the duty cycle based on stimulus frequency characteristic. For example, since we found that 11.25 Hz stimuli elicited the highest EEG amplitude, one might reduce the “on” fraction for higher-frequency stimuli to lessen visual flicker intensity, while maintaining a higher duty cycle for lower-frequency stimuli to preserve SSVEP amplitude. Additionally, a distance-adaptive adjustment mechanism could be introduced: for near-distance interaction scenarios (such as 0.4 m), dynamically lowering the duty cycle (e.g., α = 0.3–0.4) could reduce the magnitude of light intensity fluctuations over time, thereby reducing the strain of frequent lens accommodation; for mid-to far-distances (1.0, 1.8 m), the duty cycle could be maintained or slightly increased to ensure a high SNR. Furthermore, future optimizations should incorporate subjective visual fatigue assessment results (for instance, in this study the MR scenario yielded less severe diplopia) and implement dynamic brightness compensation in parallel with duty cycle adjustments. This will help ensure that fatigue is mitigated without compromising SSVEP classification accuracy or system information transfer rate, ultimately enhancing the long-term comfort and practicality of mixed reality BCI systems in near-field precise interaction scenarios (such as reading or handheld device operation).

## Data Availability

The data analyzed in this study is subject to the following licenses/restrictions: the dataset is not publicly available. Requests to access these datasets should be directed to Xiaoqian Qi, qixiaoqian@tju.edu.cn.
